# Analysis of DNA methylation-driven genes for predicting the prognosis of patients with colorectal cancer

**DOI:** 10.18632/aging.103949

**Published:** 2020-11-16

**Authors:** Boshi Fu, Cheng Du, Zhikun Wu, Mingwei Li, Yi Zhao, Xinli Liu, Huizhe Wu, Minjie Wei

**Affiliations:** 1 Department of Pharmacology, School of Pharmacy, China Medical University, Shenyang 110122, P. R. China; 2 Liaoning Key Laboratory of Molecular Targeted Anti-Tumor Drug Development and Evaluation, Liaoning Cancer Immune Peptide Drug Engineering Technology Research Center, Key Laboratory of Precision Diagnosis and Treatment of Gastrointestinal Tumors, Ministry of Education, China Medical University, Shenyang 110122, P. R. China; 3 Department of Digestive Oncology, Cancer Hospital of China Medical University, Shenyang 110042, Liaoning Province, P. R. China

**Keywords:** methylation-driven, biomarker, CRC, prognosis, pharmaceutical response

## Abstract

Aberrant promoter methylation and ensuing abnormal gene expression are important epigenetic mechanisms that contribute to colorectal oncogenesis. Yet, the prognostic significance of such methylation-driven genes in colorectal cancer (CRC) remains obscure. Herein, a total of 181 genes were identified as the methylation-driven molecular features of CRC by integrated analysis of the expression profiles and the matched DNA methylation data from The Cancer Genome Atlas (TCGA) database. Among them, a five-gene signature (POU4F1, NOVA1, MAGEA1, SLCO4C1, and IZUMO2) was developed as a risk assessment model for predicting the clinical outcomes in CRC. The Kaplan–Meier analysis and Harrell’s C index demonstrated that the risk assessment model significantly distinguished the patients in high or low-risk groups (*p*-value < 0.0001 log-rank test, HR: 2.034, 95% CI: 1.419-2.916, C index: 0.655). The sensitivity and specificity were validated by the receiver operating characteristic (ROC) analysis. Furthermore, different pharmaceutical treatment responses were observed between the high-risk and low-risk groups. Indeed, the methylation-driven gene signature could act as an independent prognostic evaluation biomarker for assessing the OS of CRC patients and guiding the pharmaceutical treatment. Compared with known biomarkers, the methylation-driven gene signature could reveal cross-omics molecular features for improving clinical stratification and prognosis.

## INTRODUCTION

Colorectal cancer (CRC) is the third most common malignancy and a leading cause of cancer-related death worldwide [1–3]. In the past decades, the survival of CRC patients has been extended progressively [4]. However, the mortality of CRC is still not satisfactory [5]. CRC is a heterogeneous cancer with a series of critical driver genomic events [6]. Multiple genetic and epigenetic changes in CRC are attracting critical attention. The gene expression profiles and the DNA methylation landscape of CRC have been widely investigated [7, 8]. Consensus molecular subtypes have been identified by Tejpar’s group for future clinical stratification and precision medicine [9]. However, subtypes were generated in a diagnostic way. The prognostic differences in patients couldn’t be reflected [10]. The prognosis of patients with CRC tends to be highly dependent on the individual. The heterogeneity of CRC made it difficult to predict prognosis and make therapeutic decisions [11]. Developing effective biomarkers is essential for improving the clinical outcome.

DNA methylation, one common epigenetic modification in eukaryotic genome [12], always exerts critical functions in regulation of gene expression and histone modifications [13]. Aberrant DNA methylation has been demonstrated as an important mechanism of oncogenic activation [14]. With the development of high-throughput sequencing for DNA methylation, genome-wide DNA methylation could be identified efficiently [15, 16]. Indeed, both hypo- and hypermethylation events in cancer have been reported [17]. The revelation of methylation map could be the key for understanding epigenetic drivers of cancer [18]. Moreover, DNA methylation was dynamic and reversible [19, 20]. The DNA methylation was regulated by DNA methylation regulators such as methyltransferase and demethylase [21]. It is still a great challenge to reveal all the molecular mechanisms and landscape of DNA methylation.

The molecular mechanism of DNA methylation has been demonstrated to be associated with colorectal tumorigenesis [22]. Relative research would be benefit for developing prognostic evaluation and clinical therapy. Bioinformatic analysis showed that some specific gene expression could be predicted by the hypo- and hypermethylation of corresponding genes exactly [23]. Such genes were identified as methylation-driven genes. Further research has reported disease-related DNA methylation-driven genes as biomarkers for early diagnosis or prognosis prediction [24].

Herein, we applied the MethylMix R package [25] to identify the methylation-driven genes in the datasets of patients with CRC from The Cancer Genome Atlas (TCGA) database. Subsequently, the potential clinical significance of these methylation-driven genes was investigated. Some specific methylation-driven genes were indicated to be associated with the prognosis by Cox regression analysis. A risk score model for survival prediction was constructed based on five-gene signature. The performance of this survival model was evaluated by Kaplan-Meier survival analysis and receiver operating characteristic (ROC) analysis. Furthermore, the difference of the pharmaceutical treatment responses between two groups classified by the survival model was investigated. The patients in the high-risk group have a higher probability of suffering clinical processive disease after chemotherapy than patients in the low-risk group. The cases after treatment by Capecitabine (Xeloda) in the high-risk group barely got complete response (CR) while the complete response rate was nearly three quarters in the low-risk group.

Consequently, methylation-driven genes could serve as a potential biomarker for predicting overall survival (OS) with clinical reference for pharmaceutical treatment response.

## RESULTS

### Identification of methylation-driven genes

**Figure 1:**
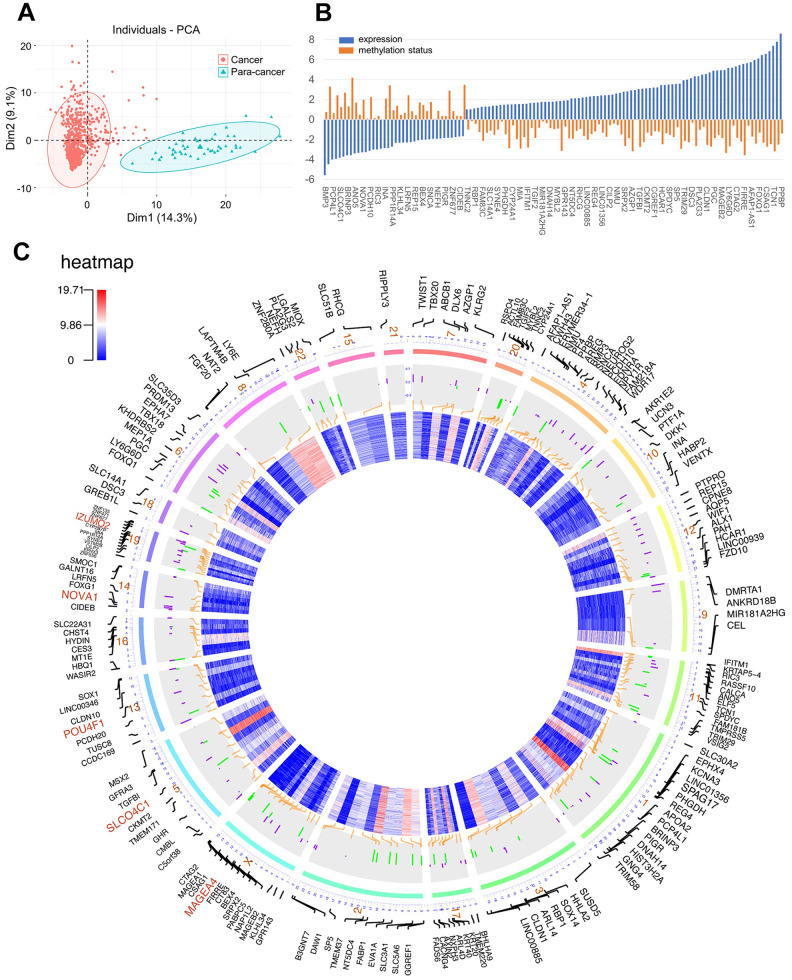
(**A**) Principal component analysis (PCA) for methylation-driven genes between solid tumor samples and normal samples; (**B**) The association of methylation status and expression; (**C**) Circos plot of DNA methylation-driven genes. From the outermost circle to the inner circle, the presentation on the map is as follows: (a) Gene symbol; (b) Chromosome location with lines deriving from specific gene locus; (c) DNA methylation by bar charts (Purple: hypermethylation, Green: hypomethylation); (d) transcriptome expression by heatmap.

In this study, the datasets of patients with CRC were all available from The Cancer Genome Atlas (TCGA). Initially, differential expression genes (DEGs) were screened out by edgeR package with the criterion of FDR < 0.05 and |log_2_FC| > 1.5 from 688 cohorts involving 638 CRC tissues and 50 normal tissues. A total of 3522 genes were identified as aberrant expressed genes in CRC (Supplementary Table 1, Supplementary Figure 1). Among them, the genes that are transcriptionally predictive by the methylation status of correlated CpG sites were identified as methylation-driven genes. The MethylMix algorithm is utilized to deriving such methylation-driven genes. The methylation status of each CpG site was quantitatively evaluated by the univariate beta mixture model. Then, the specific differentially methylated genes in cancer were identified by the comparative analysis between cancer and normal tissue. Finally, methylation-driven genes were determined by a linear regression model for association between gene expression and methylation status of its corresponding CpG sites. By this method, 181 genes were identified as methylation-driven genes from 352 specimens (308 cancer samples and 45 normal samples) within the RNA-seq data and the matched DNA methylation chip data. (Supplementary Table 2). Principal component analysis (PCA) indicated the significant difference in the expression of methylation-driven genes between cancer samples and control samples (Figure 1A). Individuals from PCA demonstrated that the over-expression of these methylation-driven genes was a significant hallmark of the cancer tumor. After chromosome location annotation, the distribution of 181 DNA methylation-driven genes were revealed. Except for Y-chromosome, DNA methylation-driven genes were distributed in all other chromosomes. In the order of the genes organized along chromosomes, the transcriptome and DNA methylome profiles were showed on the circos plots (Figure 1C). Importantly, most of these methylation-driven genes (123/181, 68.0%) were identified under negative association between methylation status and transcript level (Supplementary Figure 4) which meant that decreased methylation levels correlate with increased expression levels.

Furthermore, Two-third methylation-driven genes (118/181, 65.2%) were overexpressed in CRC tissue. They were considered as potential biomarkers for disease phenotype or clinical features. In further trials, a total of 18 genes were demonstrated to be associated with the prognosis of CRC (Supplementary Figure 7). Among them, five genes (POU4F1, NOVA1, MAGEA1, SLCO4C1, and IZUMO2) which were highlighted by the red color were recruited to construct the prediction risk model by multivariate Cox regression analysis.

### Functional analysis of the DNA methylation-driven genes

**Figure 2:**
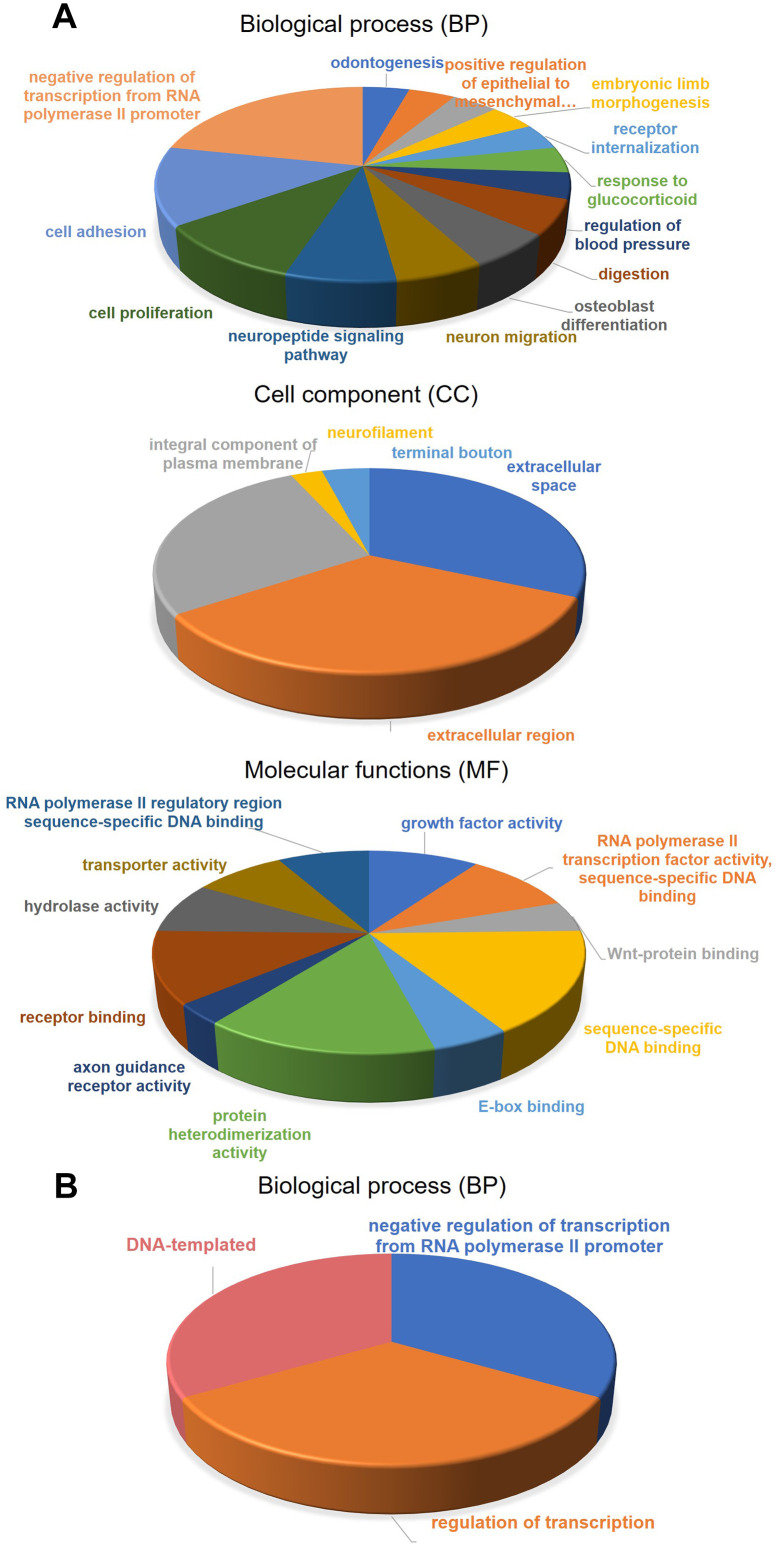
**Gene-set enrichment analysis (GSEA) for methylation-driven genes.** (**A**) molecular functions, biological process, and Cell component of 181 methylation-driven genes. (**B**) biological process of 5 genes in the risk model.

After identification of the methylation-driven genes associated with CRC, the molecular functions of these genes were investigated. As shown in Figure 2, the functional categories of 181 methylation-driven genes were defined by the gene-set enrichment analysis (GSEA) analysis (Supplementary Figure 6). The enrichment analysis showed that methylation-driven genes play critical roles in multiple categories involving 27 GO biological process (BP) terms, 5 GO cell component (CC) and 11 GO molecular functions (MF) terms (Supplementary Table 4). Overall, the functions of these genes mainly focused on the regulation of transcription. Especially, activity of RNA polymerase II was identified as the key factor. Besides that, the overexpression of these genes would promote cell adhesion and cell proliferation which are biological characteristics of cancer cells. The results were consistent with their roles in oncogenesis as methylation-driven genes. As for 18 survival-associated methylation-driven genes, the individual functional annotation table was record in supporting material (Supplementary Table 5). Finally, the biological processes regulated by the 5 genes in the risk score model were investigated by individual GO analysis (Supplementary Table 6). The results indicated that they were association with the transcription as the negative regulators of RNA polymerase II promoter (Figure 2B).

### Identification of methylation-driven genes associated with overall survival (OS)

**Figure 3:**
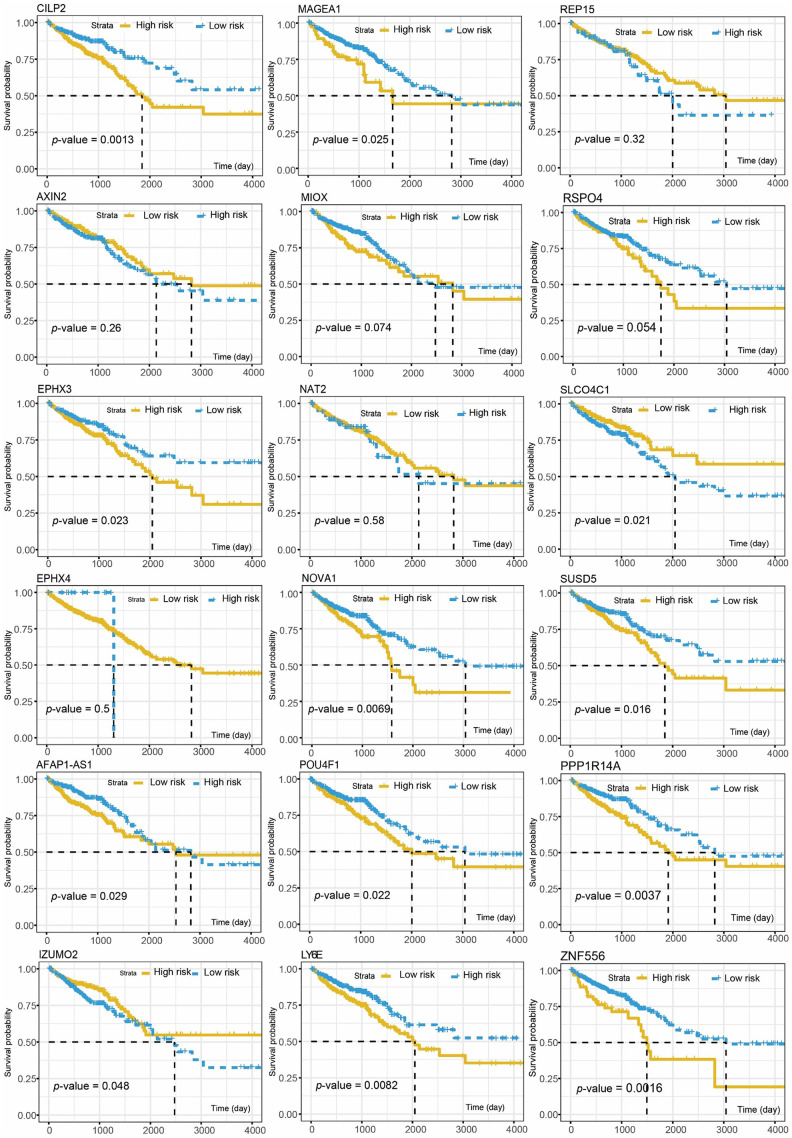
**The association between the OS and individual gene expression of the 18 survival-associated genes.**

**Figure 4:**
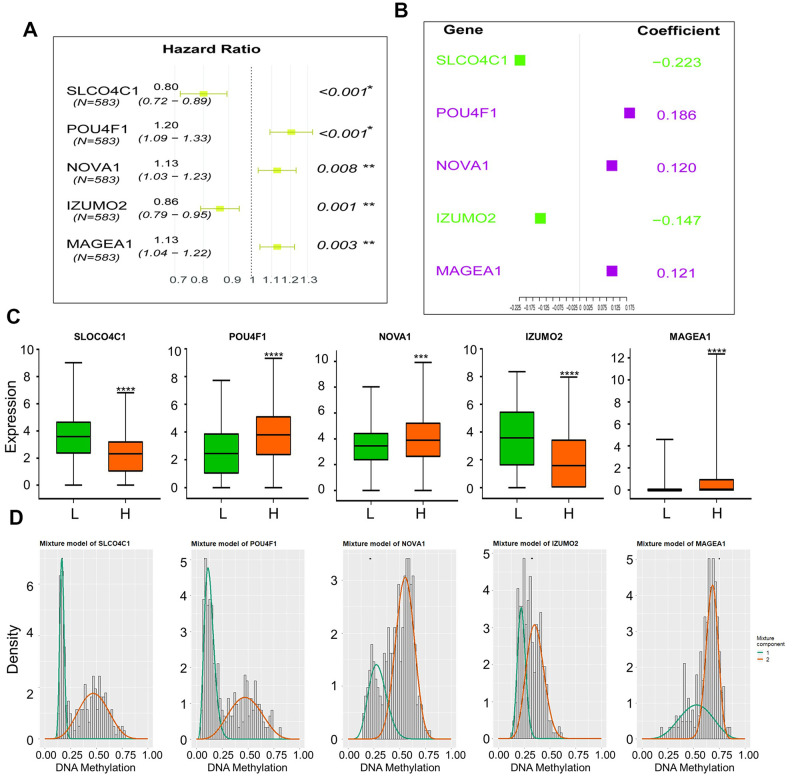
(**A**) Hazard Ratio of genes form the survival model; (**B**) Coefficient of genes form the survival model; (**C**) Expression of five genes in high-risk and low-risk groups, (“L”: low-risk group, “H”: high-risk group), Mann–Whitney test was used to evaluate the differences between the two groups,****: p-value < 0.0001, ***: p-value = 0.0002; (**D**) Mixture models of five genes.

A total of 581 patients diagnosed with CRCs were included in the survival analysis. The median age was 68 years (range, 30–89 years). The information of TNM classification was displayed in Table 1. The Cox proportional hazard regression analysis was employed to investigate the association between methylation-driven genes and clinical survival time in the CRC patients. Initially, a total of 18 genes among the methylation-driven genes were identified to be significantly associated with OS of patients with CRCs (*p-*value < 0.005) by univariate Cox regression analysis (Table 2). And the significant analysis for association between the OS and expression of the individual gene was investigated by log-rank test (Figure 3). Focused on these genes, multivariate Cox regression analysis was further performed to construct a scoring model for survival prediction. By the Akaike Information Criterion (AIC), 9 genes (ZNF556, CILP2, NAT2, REP15, SUSD5, MIOX, RSPO4, PPP1R14A and LY6E) were eliminated (Supplementary Table 4). Then, step elimination optimization was proposed to ensure all the genes in models were statistically significant (*p*-value < 0.01, Table 3) Finally, the expression of five genes (POU4F1, NOVA1, MAGEA1, SLCO4C1, IZUMO2) were defined as the index to obtain the risk assessment model (Table 3, Figure 4A, 4B). The risk scoring formula was defined as follows: RiskScore=POU4F1×1.2040333+NOVA1×1.1272212+MAGEA1×1.1276608+SLCO4C1×0.8003405+ IZUMO2×0.8645822.

**Table 1:** Clinicopathological characteristics of CRC patients from TCGA.

**Variables**	**Patients**					
	**Total**		**Training dataset**		**Test dataset**	
	**No.**	**%**	**No.**	**%**	**No.**	**%**
**Gender**						
Female	310	53.91%	221	54.84%	89	51.45%
Male	265	46.09%	181	44.91%	84	48.55%
**Age at diagnosis**						
Median	68		68		67	
Range	31-90		34-90		37-90	
>60	392	68.17%	277	68.73%	117	67.63%
<61	183	31.83%	126	31.27%	52	30.06%
**TNM stage (T)**						
T1	19	3.30%	9	2.23%	10	5.78%
T2	102	17.74%	66	16.38%	36	20.81%
T3	393	68.35%	282	69.98%	111	64.16%
T4	61	10.61%	45	11.17%	16	9.25%
**TNM stage (N)**						
N0	327	56.87%	222	55.09%	105	60.69%
N1	142	24.70%	97	24.07%	45	26.01%
N2	106	18.43%	83	20.60%	23	13.29%
**TNM stage (M)**						
M0	435	75.65%	303	75.19%	132	76.30%
M1	82	14.26%	58	14.39%	24	13.87%
Mx	58	10.09%	41	10.17%	17	9.83%

**Table 2:** The results of univariate Cox analysis.

**Gene**	**HR**	**z**	**95%CI**	***p* value**
SLCO4C1	0.8560	-2.9084	0.86 [0.77, 0.95]	0.0036
ZNF556	1.1087	2.8937	1.11 [1.03, 1.19]	0.0038
MAGEA1	1.1157	2.7109	1.12 [1.03, 1.21]	0.0067
AFAP1.AS1	1.0764	2.6837	1.08 [1.02, 1.14]	0.0073
CILP2	1.1273	2.6260	1.13 [1.03, 1.23]	0.0086
NAT2	0.8921	-2.5460	0.89 [0.82, 0.97]	0.0109
REP15	0.8971	-2.4593	0.90 [0.82, 0.98]	0.0139
POU4F1	1.1202	2.4028	1.12 [1.02, 1.23]	0.0163
EPHX4	0.8765	-2.2504	0.88 [0.78, 0.98]	0.0244
MIOX	1.1170	2.1943	1.12 [1.01, 1.23]	0.0282
SUSD5	1.1184	2.1421	1.12 [1.01, 1.24]	0.0322
RSPO4	1.0843	2.1218	1.08 [1.01, 1.17]	0.0339
IZUMO2	0.9140	-2.1136	0.91 [0.84, 0.99]	0.0346
NOVA1	1.1012	2.0521	1.10 [1.00, 1.21]	0.0402
EPHX3	1.1272	2.0510	1.13 [1.01, 1.26]	0.0403
AXIN2	0.8946	-2.0120	0.89 [0.80, 1.00]	0.0442
PPP1R14A	1.1511	1.9970	1.15 [1.00, 1.32]	0.0458
LY6E	1.1462	1.9954	1.15 [1.00, 1.31]	0.0460

**Table 3:** The results of multivariate Cox analysis.

**Gene**	**coef**	**exp(coef)**	**se(coef)**	**z**	**Pr(>|z|)**
SLCO4C1	-0.2227	0.8003	0.0556	-4.0087	0.000061
MAGEA1	0.1201	1.1277	0.0412	2.9166	0.003539
POU4F1	0.1857	1.2040	0.0508	3.6529	0.000259
IZUMO2	-0.1455	0.8646	0.0459	-3.1668	0.001541
NOVA1	0.1198	1.1272	0.0451	2.6533	0.007972

The median value of the RiskScore (0.961639) was defined as the intergroup cut-off value. According to this value, the specimens could be classified into high-risk group and low-risk group. Further, Mann–Whitney testing indicated significant differential expression of individual genes in the risk model between the high-risk group and the low-risk group (Figure 4C). The correlation between expression of the five genes and the methylation status was verified by Pearson correlation coefficient (Supplementary Figure 2, Supplementary Table 3). However, there is no evidence for association between OS and methylation pattern of these genes. (Supplementary Figure 9) The methylation β mixed model of these genes was shown in Figure 4D. The black horizontal line was the scale for indicating the methylation status in normal specimens. The curves were fitted with the subgroups of differential methylation in the cancer group, and it could represent the trend of methylation distribution in CRC tissues.

### Predictive performance of the methylation-driven gene signature

**Figure 5:**
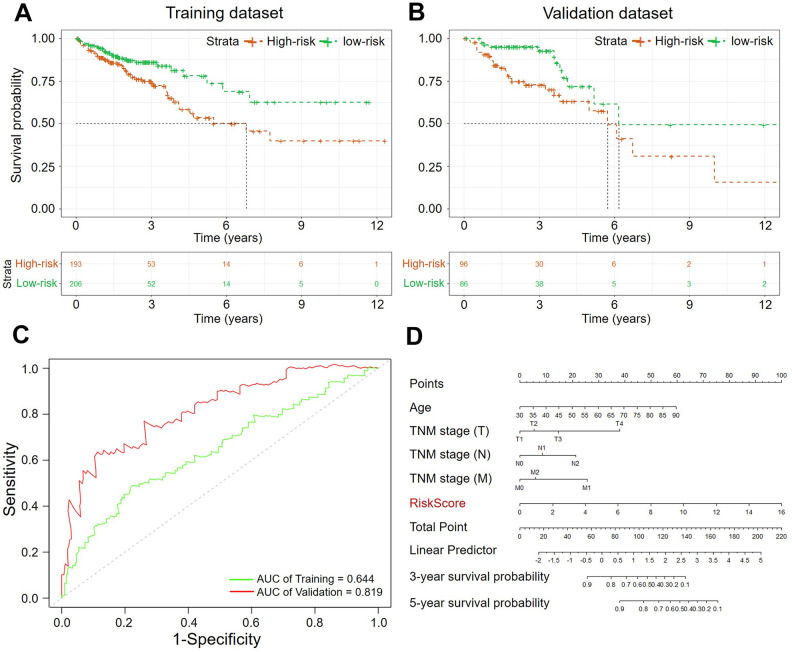
**The Kaplan–Meier curves of the OS for high-risk and low-risk.** (**A**) Training dataset (*p*-value < 0.005); (**B**) Validation dataset (*p*-value < 0.005); (**C**) ROC analysis of sensitivity and specificity (Green: Training dataset, Red: Validation dataset; (**D**) Nomogram of clinicopathological characteristics and RiskScore.

**Figure 6:**
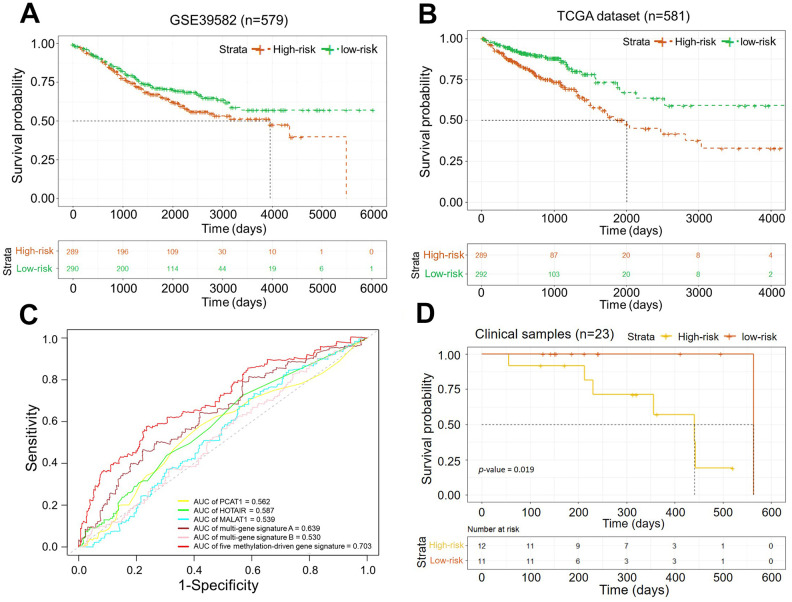
(**A**) Kaplan–Meier curves of the OS in dataset GSE39582 (*p*-value < 0.05); (**B**) Kaplan–Meier curves of the OS in the dataset from TCGA (*p*-value = 0.0001); (**C**) ROC curves of five methylation-driven gene signature and other known biomarkers for prognosis in CRCs. (**D**) Kaplan–Meier curves of the OS in clinical samples (*p*-value = 0.019).

The 581 specimens were separated randomly into a training dataset (402 samples) and a validation dataset (173 specimens) with a ratio of 3:1. The Kaplan–Meier analysis was performed to evaluate the predictive value of this risk assessment model in the prognosis. The *p*-value from log-rank tests and hazard ratios (HRs) from the Cox regression analysis indicated that our hazard model based on five methylation-driven genes was significantly associated with the OS of patients with CRC (Training dataset: *p-*value < 0.005, HR: 2.543, 95% CI: 1.291–5.008; Validation dataset: *p-*value < 0.005, HR: 2.075, 95% CI: 1.421–3.029, Figure 5A). The sensitivity and specificity of our prognosis risk assessment model were verified by the receiver operating characteristic (ROC) curve (Figure 5C). The AUC values of both datasets (training dataset: 0.644, testing dataset: 0.819) indicated that our risk assessment model could be an effective marker for predicting the prognosis of CRC. For all the specimens, the cohort was classified into two groups according to the median value of the RiskScore. The Kaplan–Meier curve showed that our five-gene signature could accurately distinguish high- and low-risk patients with CRC significantly *(p*-value < 0.0001, HR: 2.034, 95% CI: 1.419-2.916). The Harrell’s C index revealed a value of 0.655. This meant that a significant difference between the high-risk group and the low-risk group. The mean OS of patents in the high-risk group was 2003 days while the mean OS in the low-risk group was identified as NA because of the expectable good prognosis. By the same way, the effectiveness of five-gene model was validated in an independent GEO cohort (GSE39582, Figure 6A). Specifically, the result from the multivariate Cox analysis for the clinical characteristics (age, sex, grade, and TNM classification) and RiskScore demonstrated the independence of our risk assessment model (Table 4). Moreover, the effect of risk factors for predicting survival could be evaluated by the nomogram (Figure 5D, Supplementary Figure 3). Compare to clinical characteristics including age, gender and TNM classification, RiskScore based on five methylation-driven gene signature occupied maximum proportion in the pointing system. It means that RiskScore played the most important role in predicting system.

**Table 4:** The predictive values of related clinical characteristics and RiskScore.

**Clinical Characteristic**	**coef**	**exp(coef)**	**se(coef)**	**z**	**Pr(>|z|)**
TNM stage (T)	0.7595	2.1372	0.2029	3.742	0.000182
TNM stage (N)	0.5062	1.6590	0.1187	4.266	0.000020
TNM stage (M)	0.2624	1.3001	0.1248	2.102	0.035549
age	0.0334	1.0340	0.0081	4.127	0.000037
gender	0.0287	1.0291	0.1880	0.153	0.878587
RiskScore	0.2500	1.2841	0.0436	5.741	9.39E-09

Finally, the prognostic value of our methylation-driven gene signature was validated in 23 patient tumor samples provided by Cancer Hospital of China Medical University, Liaoning Cancer Hospital and Institute. The detail of clinical information was list in the supporting information (Supplementary Table 8). Total RNA had been extracted for the solid tissues and gene expression profiles were detected by qPCR (Supplementary Figure 10 and Supplementary Tables 7, 9). Similarly, the risk score from the 5-gene signature divided patients into high- and low-risk groups. Kaplan–Meier curves indicated significant differences between the two groups (log-rank test, *p*-value < 0.05; Figure 6D) All the results demonstrated that the prognosis risk assessment model based on the five-gene signature could be an independent applicable predictor for prognosis in evaluation in CRC patients.

### Comparison of prognosis model based on methylation-driven genes with other known biomarkers

In recent years, several prognostic biomarkers in CRC based on molecular features such as aberrant expressions were developed. For instance, high expression of MALAT1 suggested poor prognosis in CRC patients [26]. HOTAIR could be identified as a negative prognostic factor both in primary tumors and blood of CRC patients [27, 28]. PCAT-1, identified as prostate cancer–associated ncRNA transcripts 1, was also demonstrated to be associated with worse prognosis clinical outcomes in CRC [29]. Furthermore, multi-gene signatures were also developed as novel prognosis biomarkers by multivariate Cox analysis. H. Chen, Sun, et al. [30] reported a seven-gene signature (PPIP5K2, PTPRB, NHLRC3, PRR14L, CCBL1, PNPO, and ZDHHC21) as prognostic biomarkers by analyzing a gene microarray of 64 specimens. Zhuang Li, et al. [31] developed a five-gene signature (KIF15, NAT2, GPX3, SCG2, and CLCA1) for predicting the OS of CRC patients in two independent GEO cohorts.

Herein, the sensitivity and specificity of above known biomarkers and our risk assessment model were verified uniformly by ROC analyses. The data from TCGA program (COAD, READ) were used as the validation dataset. As a result, our risk assessment model based on five methylation-driven genes showed effective and reliable performance (Figure 5C). The signature of five methylation-driven genes was demonstrated to be an effective predictor for OS of patients with CRC.

### Pharmaceutical treatment response of patients from the risk assessment model

**Figure 7:**
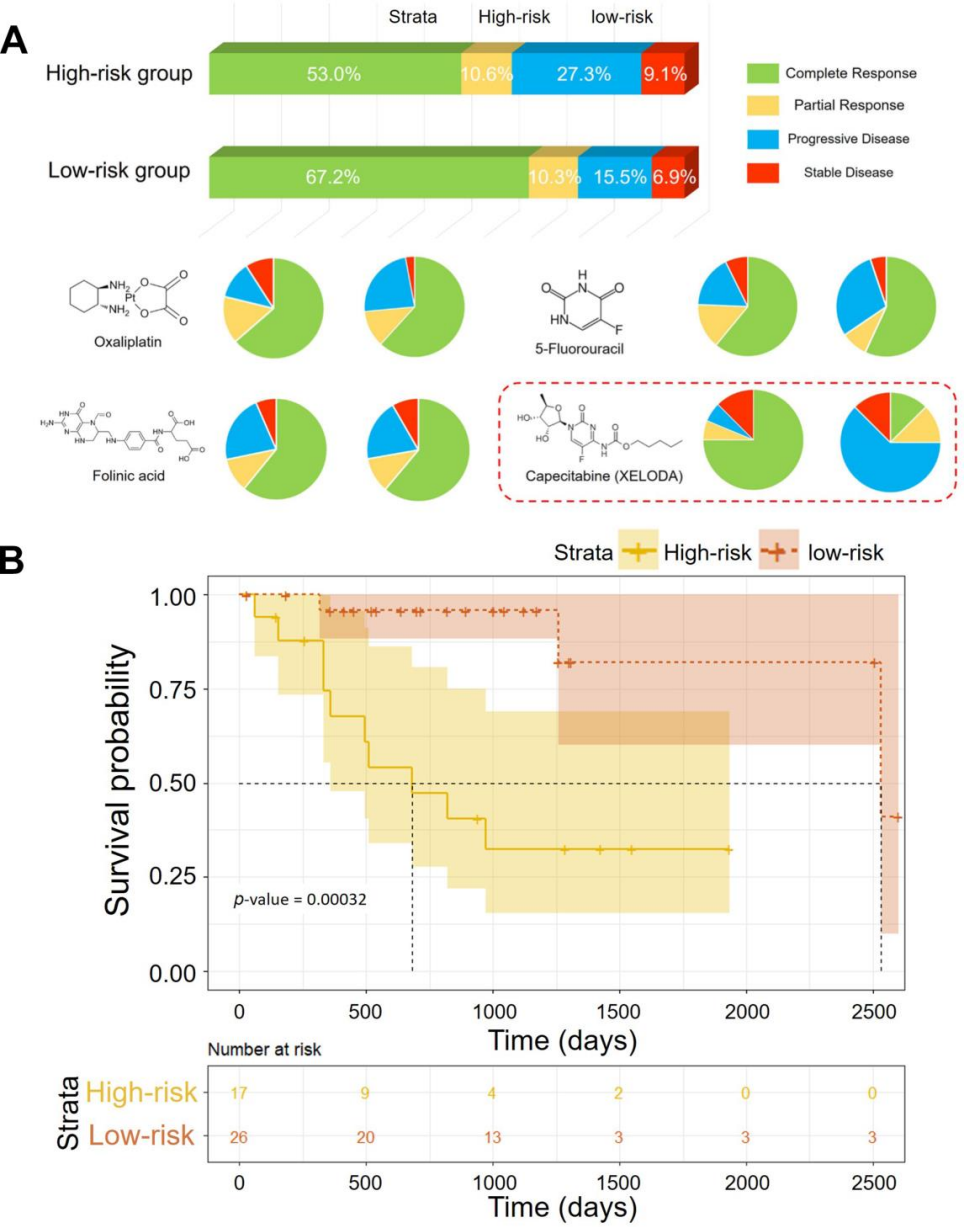
(**A**) Pharmaceutical treatment responses of patients in the low-risk group and the high-risk group. (**B**) Kaplan–Meier curves of the OS of patients treated with XELODA in TCGA (*p*-value = 0.00032).

More than the prognosis, the differences in pharmaceutical treatment response between the high-risk group and the low-risk group were observed. the clinical information of pharmaceutical therapy events and the matched treatment responses were also available in TCGA database. A total of 828 events of pharmaceutical therapy from 232 patients with CRC were recorded. According to the Riskscore, the patients were classified in the high-risk group and the low-risk group. Then, statistical analysis revealed the difference of treatment responses between two groups. Generally, treatment response of patients in the low-risk group was better of the two groups. The proportion of patients with complete response (CR), partial response (PR), progressive disease (PD) and stable disease (SD) and were 53.0%, 10.6%, 27.3% and 9.1% in the high-risk group and 67.2%, 10.3%, 15.5% and 6.9% in the low-risk group, respectively. Two thirds of cases from the low-risk group achieved clinical complete response after chemoradiotherapy vs 53% in the high-risk group. Moreover, patients in the high-risk group had higher rates of suffering clinical progressive diseases after chemotherapy (27.3% vs 15.5%). Treatment responses of specific drugs were displayed by pie charts (Figure 7A). Among them, significant difference in CR rates of treatment by capecitabine (Xeloda) was observed. The CR rates were 75% in the low-risk group while 12.5% in the high-risk group. Different efficacy of capecitabine (Xeloda) was reflected. We extracted the patients treated with Capecitabine (Xeloda) from the TCGA datasets and carried out systematic survival analyses. The association between the OS and individual gene was investigated (Supplementary Figure 8.). Consistently, the risk scores from the methylation-driven gene signature indicated the risk of prognosis. As shown in Figure 7B, the significant different of OS in two group was demonstrated by the log-rank test (*p*-value < 0.00032).

## DISCUSSION

Critical driver genomic events that contribute to oncogenesis are important mechanisms of the developmental processes of CRC. Molecular signatures such as aberrant expression, mutation, and methylation have been indicated to be various biomarkers. For example, oncogenes like PCAT-1[29], MALAT1 [26], and NDRG4 [32] which associated with progression in pan-cancer had also been identified as prognostic biomarkers in CRC. High HOTAIR expression in primary tumors or in the blood of CRC patients was associated with poor prognosis [28]. With the development of high through-put technology, it’s convenient to get gene expression profiles. The clinical values of multi-gene signatures were widely investigated. However, it is difficult to evaluate the prognostic value of biomarkers uniformly because of the differences in analysis methods and data sources. As potential effective and convenient biomarkers, DEG-based signature is deserved to be further investigated.

DNA methylation was an important epigenetic event in driving oncogenesis. DNA methylation on specific sites could regulate corresponding gene expression. Such gene was identified as methylation-driven gene. Cancer-specific methylation-driven genes were demonstrated with vital clinical value [33].

In this study, a total of 181 genes were identified as CRC-specific methylation-driven genes. Functional enrichment analysis indicated that these genes would contribute to the cell cellular transformation and aberrant translation in CRC. Then, 18 genes among the 181 methylation-driven genes were demonstrated to be associated with the OS of CRC patients. Based on these survival-related genes, the five-gene signature (SLCO4C1, MAGEA1, POU4F1, IZUMO2, and NOVA1) was developed as a prognostic prediction biomarker for CRC (Supplementary Figure 5). In previous research, SLCO4C1 promoter methylation have been identified as prognostic biomarker for prostate cancer [34]. NOVA1 was proven to be a crucial factor promoting telomerase activity in cancer cells [35]. The aberrant expression of MAGE-A1 and POU4F1 was also reported to be associated with multiple diseases [36, 37]. In this study, there five-gene signature construct a risk assessment model for predicting the prognosis of patients with CRC. Genes ontology annotation indicated that they could increase the translation by negative regulation of RNA polymerase II promoter. The sensitivity and specificity of the model were verified by Harrell’s C index and receiver operating characteristic (ROC) analysis. The log-rank test demonstrated that there was significant difference between the high-risk group and the low-risk group (*p-*value < 0.0001, log-rank). Multivariate Cox analysis and nomogram indicated that the five-gene signature was an independent prognostic biomarker for CRC. Thus, the signature of the five CRC-specific methylation-driven indicators should be an effective prognostic candidate biomarker. However, the prognostic value of this assessment model needs to be further verified by clinical trials in the future.

More than other biomarkers based on DEG, the expression of methylation-driven genes was associated with the DNA methylation status of corresponding sites. Correlation between the expression of five genes and methylation status was demonstrated by Pearson correlation coefficient. According to the expression, the foregoing hyper-or hypomethylation events could be inferenced. This could be convenient for assessing the cross-omics molecular features of tumor tissue.

Furthermore, DNA methylation has been reported as a possible driver of therapeutic resistance. Importantly, the difference in pharmaceutical treatment response between the high-risk group and the low-risk group was observed. Patients in the high-risk group are more likely to suffer clinical progressive diseases after chemotherapy. This might be one reason of the shorter OS. Especially, Capecitabine (XELODA) hardly got complete response from patients in the high-risk group. Similarly, DNA methylation was reported to lead to capecitabine resistance in mesothelioma [38]. Although, the underlying mechanism in CRC remains unclear, some reference suggestions for clinical therapy will be provided by the signature of five methylation-driven genes.

## CONCLUSIONS

In conclusion, the methylation-driven genes associated with CRC were identified by analyzing gene-expression profiles and corresponding DNA methylation data which are available at the TCGA database. Among these genes, a total of 18 genes were indicated to be significantly associated with the OS of patients. A risk assessment model for predicting the prognosis was constructed based on five methylation-driven genes. After verification, the signature of five methylation-driven genes was demonstrated to be an independent prognostic biomarker for CRC. Further clinical response evaluation indicated that the patients classified as high risk would always be with worse pharmaceutical treatment response. Our findings would provide a novel biomarker for improving the clinical outcome of CRC patients and new insights into aberrant DNA methylation in CRC.

## MATERIALS AND METHODS

### DNA methylation data and gene expression data preprocessing

The data for DNA methylation, RNA-Sequencing (RNA-Seq) and corresponding clinical information were downloaded for The Cancer Genome Atlas (TCGA). The DNA methylation data had been generated from the Illumina Infinium Human Methylation 450K platform. The DNA methylation status was evaluated by β value after preprocessing. RNA sequencing data were normalized by the “edgeR” package in R software. Specimens used for survival analysis required complete survival information. Clinical samples were provided by Cancer Hospital of China Medical University. Liaoning Cancer Hospital and Institute.

### Analysis of differentially expressed genes

After preprocessing, differentially expressed genes (DEGs) were screened out by means of the quasi-likelihood F test as per its instruction. We as a result selected the differential genes with |logFC| > 1.5 and *p*-value < 0.05 for further research.

### Identification of methylation-driven genes

The methylation-driven genes were identified by the "methylmix" package. The specimens used for analyzing contained both DNA methylation chip data and corresponding RNA-seq data. For investigating the correlation between methylation status and expression (cis-regulation) of specific genes, the methylation status of each gene was evaluated by a single-variable β mixed model based on Bayesian information criterion (BIC) and the Wilcoxon rank sum test. Linear regression was used to simulate the correlation between the methylation status and expression profile of each gene. The genes were identified as methylation-driven genes if the correlation coefficient was low than -0.4 and |DM-values (differential methylation values)|>0. Chromosome positions of DNA methylation-driven genes were visualized by “OmicCircos” (R package). The methylation status and the copy number variation were displayed synchronically.

### Gene Ontology enrichment analysis of DNA methylation-driven genes

The “clusterProfiler” package was used to perform gene set enrichment analysis of gene-set enrichment analysis (GSEA) on DNA methylation-driven genes with *p*-value-Cutoff was set at 0.05 as the filter criteria. After that, “enrichplot” package was operated to visualize holistic authentic results.

### Cox regression analysis

The Cox regression analysis was conducted by using “survival” package. We performed univariate Cox regression analysis of all DNA methylation-driver genes in TCGA following removing censored data, from which eighteen genes were identified for they were significantly associated with survival (*p* value < 0.05). After that, multivariate cox regression analysis was performed to explore the association between the survival time of patients and predictor variables thus building a risk model. Finally, five genes were obtained for risk assessment and risk assessment formula was:

$$Risk\;score=\sum_{\text{i}=1}^{\text{n}}{\text{Coef}}_{\text{i}}{\text{*Exp}}_{\text{i}}$$

where Coefi belongs to the coefficient of a specific model gene, and Expi pertains to the expression level of each selected gene. The Harrell’s C index was calculated by the “survcomp” package.

### Survival analysis

The prognostic value of risk score based on methylation-driven genes on OS of patients with CRC was investigated by Kaplan–Meier analysis with log-rank test (Mantel–Cox) using the “survival” package. ROC curves were built by the “survivalROC” package. Nomogram analysis for independence of biomarkers were performed with “rms” package.

### Ethics statement

Ethical approval has been obtained by The Cancer Genome Atlas. (TCGA, https://tcga-data.nci.nih.gov/tcga/). The expression array database (GSE39582) were downloaded from the Gene Expression Omnibus (GEO, https://www.ncbi.nlm.nih.gov/geo/).

## Supplementary Material

Supplementary Figures

Supplementary Table 1

Supplementary Table 2

Supplementary Tables 3 and 4

Supplementary Table 5

Supplementary Tables 6, 7, 8 and 9
