# Supplementary material for: Analysis of DNA methylation-driven genes for predicting the prognosis of patients with colorectal cancer
Source: Aging (Albany NY). 2020 Nov 16;12(22):22814–39. doi: 10.18632/aging.103949 (PMC7746389; doi:10.18632/aging.103949)
Supplement: Supplementary Figures [file aging-12-103949-s001..pdf]

SUPPLEMENTARY FIGURES

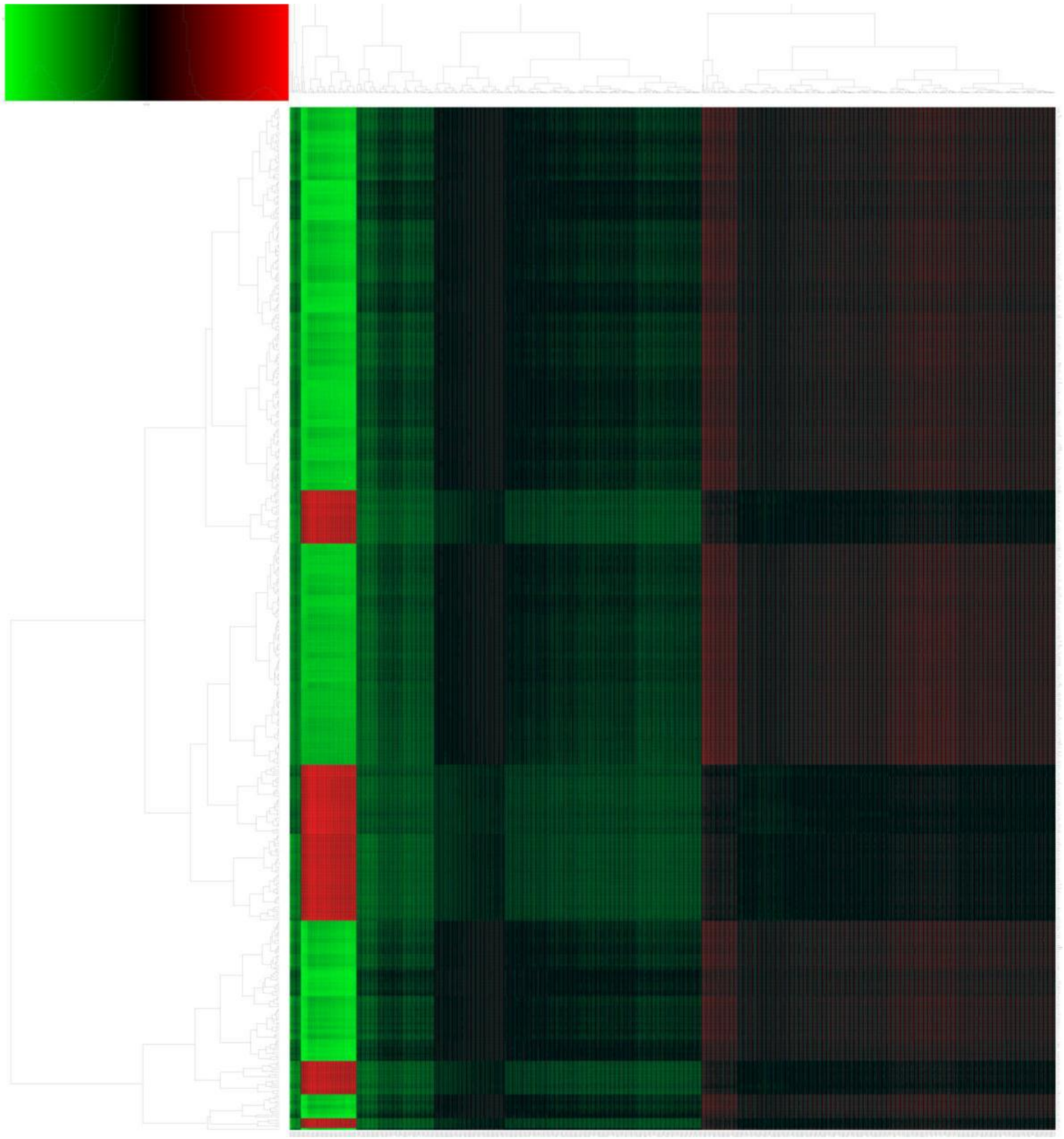

Supplementary Figure 1. Hierarchical cluster dendrogram of differentially expression genes from the TCGA dataset.

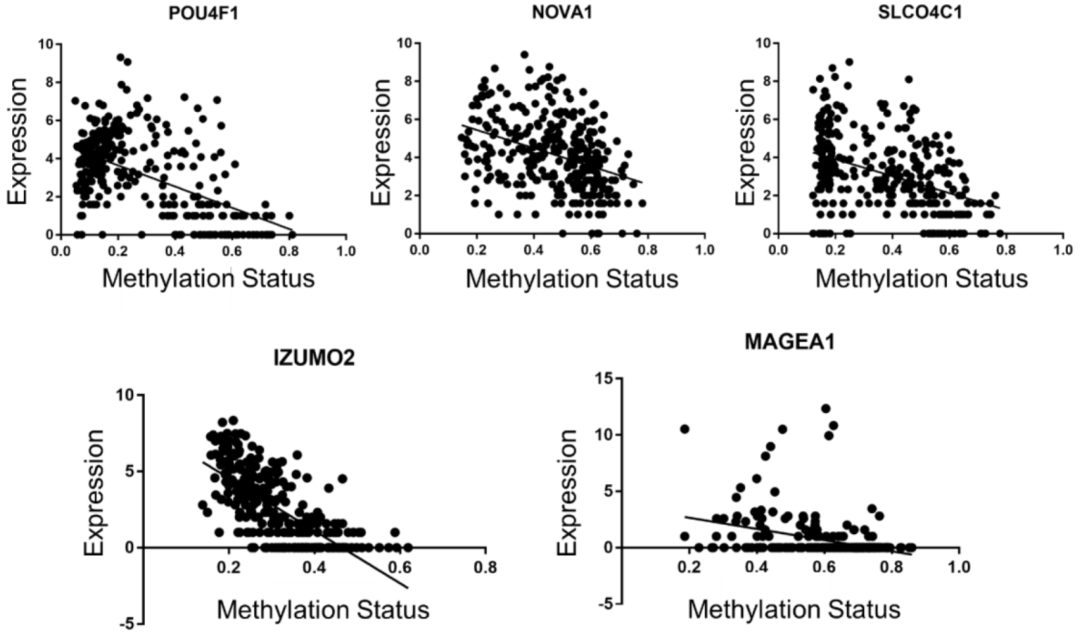

Supplementary Figure 2. The correlation between expression of the five genes and the methylation status was verified by Pearson correlation coefficient.

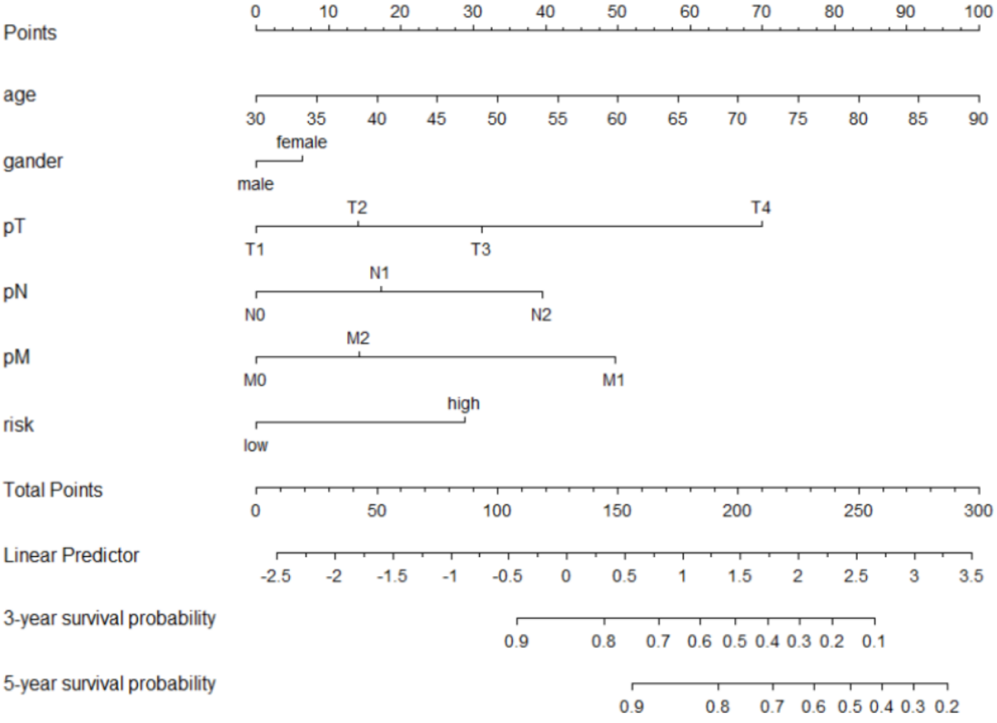

Supplementary Figure 3. Nomogram of clinicopathological characteristics and RiskRank.

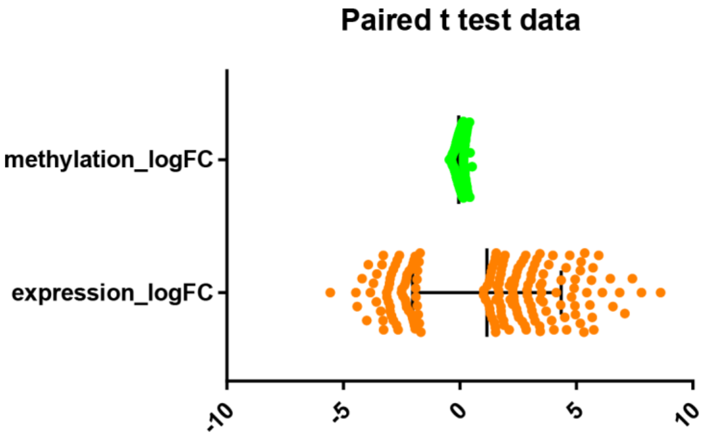

Supplementary Figure 4. Association between methylation status and expression.

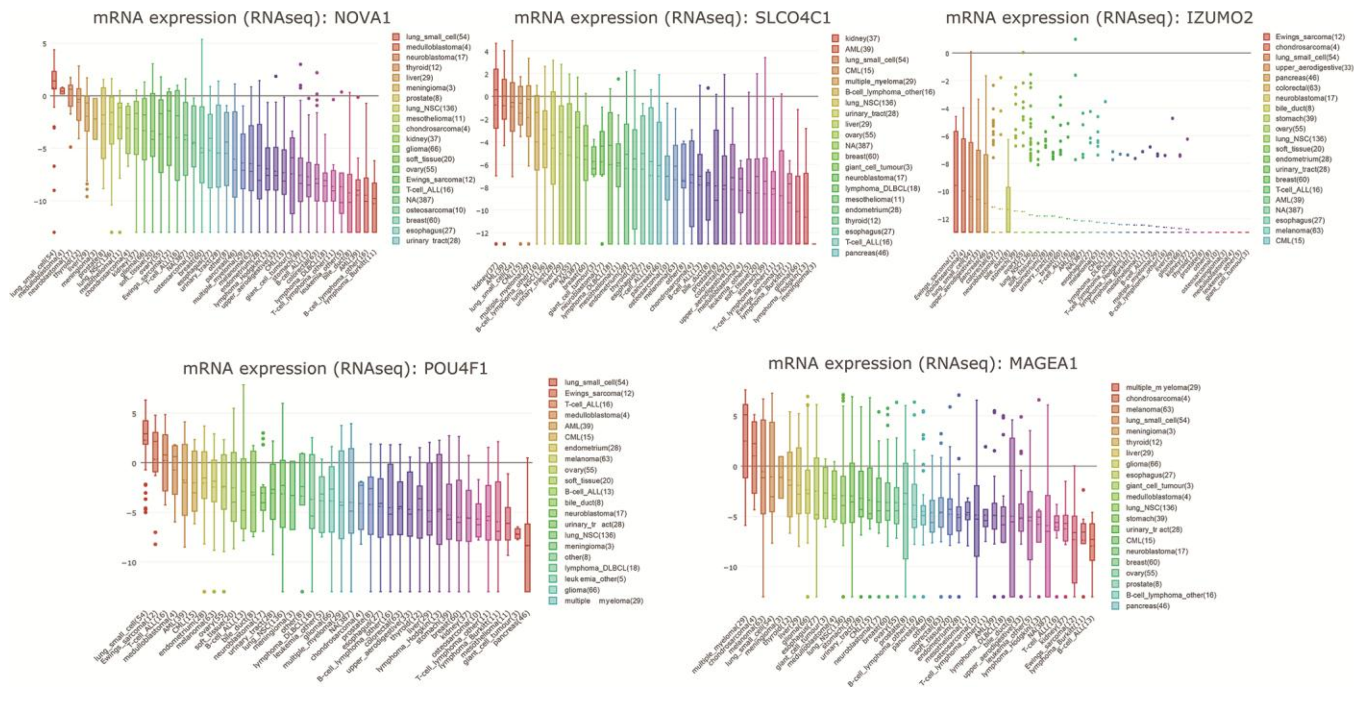

Supplementary Figure 5. Expression of 5 candidate genes in risk model in cell lines.

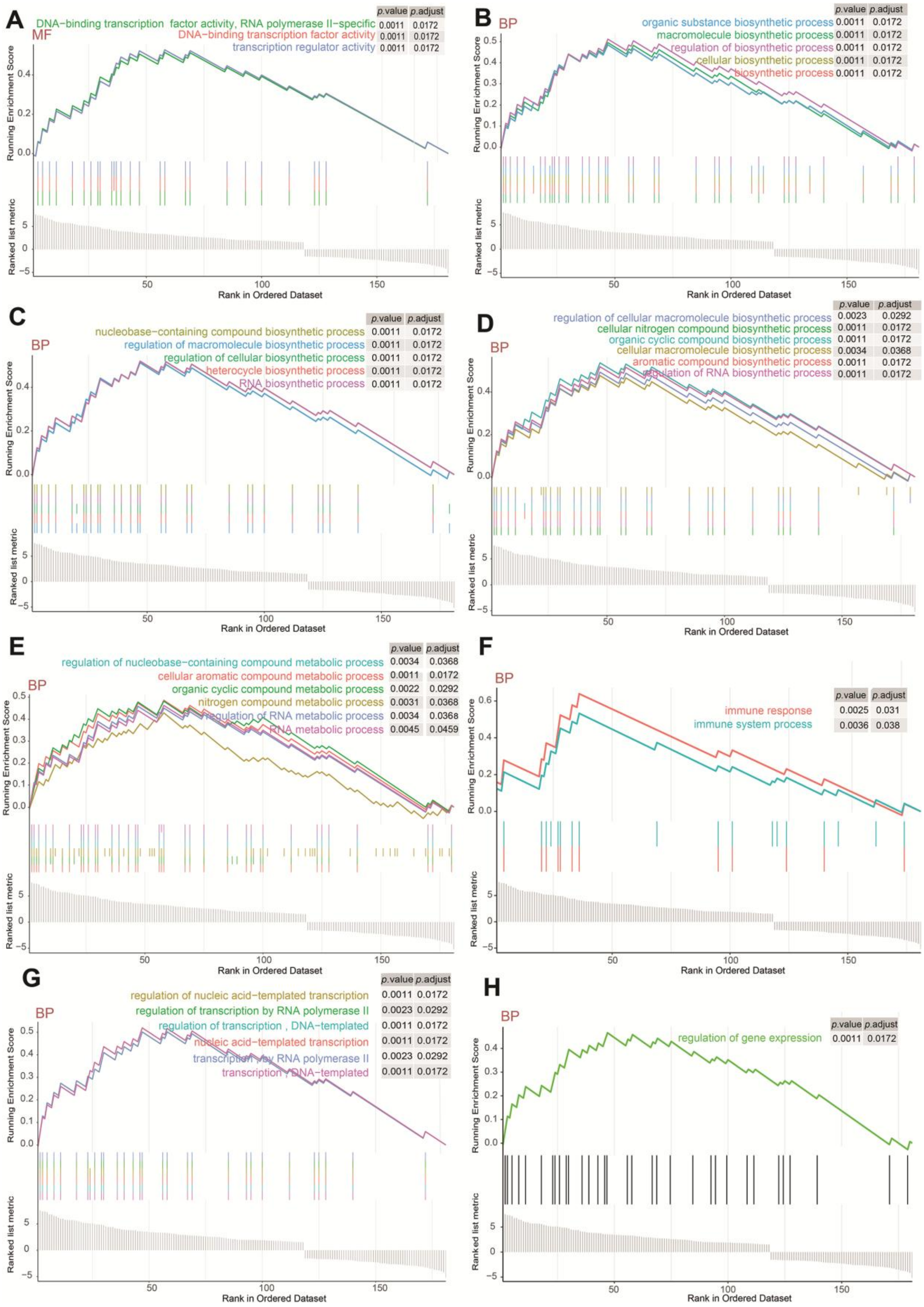

Supplementary Figure 6. Gene-set enrichment analysis (GSEA) for 181 methylation-driven genes. (**A**) Molecular functions; (**B**–**H**) Biological process (BP).

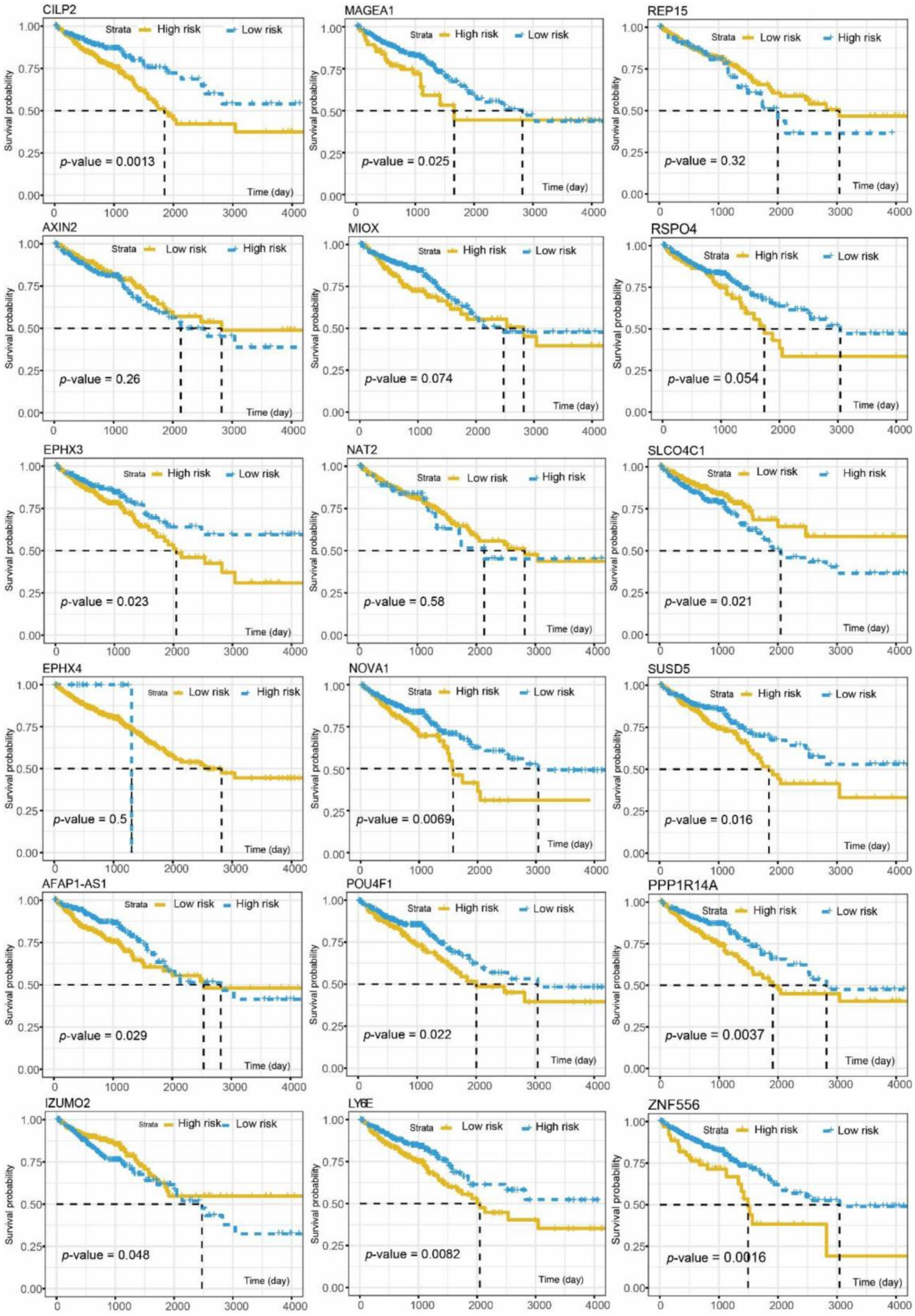

Supplementary Figure 7. The association between the OS and individual gene expression of the candidate genes.

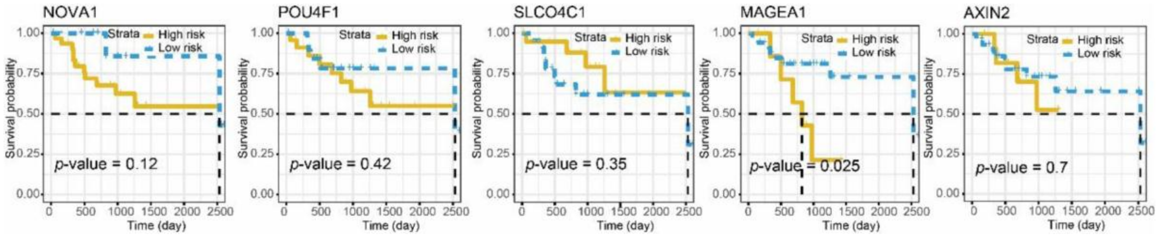

Supplementary Figure 8. The association between the OS of patients who had treated with Capecitabine (Xeloda) and individual gene.

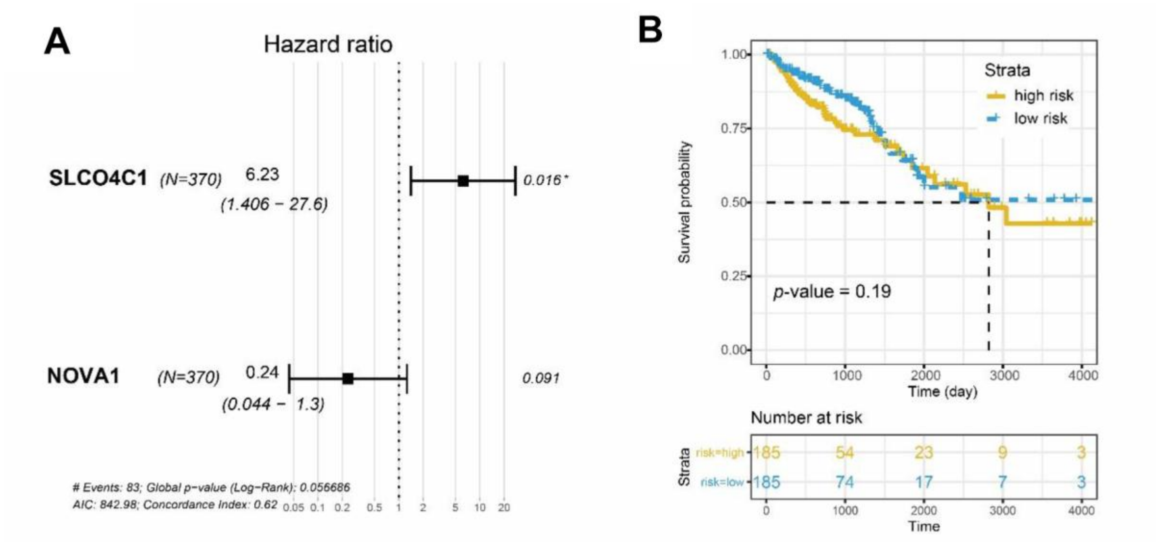

Supplementary Figure 9. The association between OS and methylation status of 5 genes. (**A**) Hazard Ratio of genes form the survival model; (**B**) Survival curve.

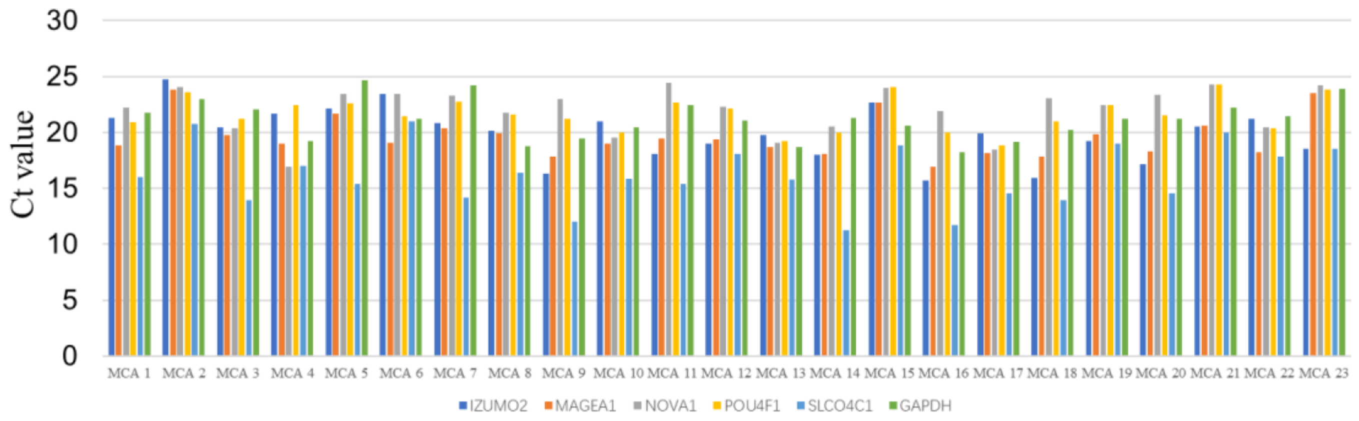

Supplementary Figure 10. Ct value from q-pcr in 23 clinical samples.
