# Supplementary material for: Analysis of DNA methylation-driven genes for predicting the prognosis of patients with colorectal cancer
Source: Aging (Albany NY). 2020 Nov 16;12(22):22814–39. doi: 10.18632/aging.103949 (PMC7746389; doi:10.18632/aging.103949)
Supplement: Supplementary Tables 3 and 4 [file aging-12-103949-s004..pdf]

SUPPLEMENTARY TABLES

Supplementary Table 3. Pearson correlation coefficient.

Gene	Pearson r	95% confidence interval	R squared	*P* value	*P* value summary
POU4F1	-0.5424	-0.6168 to -0.4584	0.2942	<0.0001	****
NOVA1	-0.3723	-0.4648 to -0.2719	0.1386	<0.0001	****
SLCO4C1	-0.4212	-0.509 to -0.3247	0.1774	<0.0001	****
IZUMO2	-0.7123	-0.7633 to -0.6525	0.5074	<0.0001	****
MAGEA1	-0.358	-0.4517 to -0.2565	0.1282	<0.0001	****

Supplementary Table 4. 9 genes selected in multi-variation cox regression through Akaike Information Criterion.

	coef	exp(coef)	se(coef)	z	*p*-value
SLCO4C1	-0.22759	0.79645	0.05719	-3.979	6.91E-05
MAGEA1	0.10437	1.11001	0.04248	2.457	0.01402
AFAP1.AS1	0.06345	1.06551	0.02872	2.21	0.02714
POU4F1	0.21784	1.24339	0.05376	4.052	5.08E-05
EPHX4	-0.0948	0.90955	0.06291	-1.507	0.13185
IZUMO2	-0.12456	0.88288	0.04751	-2.622	0.00875
EPHX3	0.11068	1.11704	0.06058	1.827	0.06771
NOVA1	0.13369	1.14304	0.04786	2.793	0.00522
AXIN2	-0.1214	0.88568	0.06292	-1.929	0.05367
