# Supplementary material for: Analysis of DNA methylation-driven genes for predicting the prognosis of patients with colorectal cancer
Source: Aging (Albany NY). 2020 Nov 16;12(22):22814–39. doi: 10.18632/aging.103949 (PMC7746389; doi:10.18632/aging.103949)
Supplement: Supplementary Tables 6, 7, 8 and 9 [file aging-12-103949-s006..pdf]

SUPPLEMENTARY TABLES

Supplementary Table 6. Gene ontology for 5 survival-associated methylation-driven genes.

	IZUMO family member 2(IZUMO2)
GOTERM_BP_DIRECT	regulation of transcription, DNA-templated,
GOTERM_CC_DIRECT	nucleus, integral component of membrane,
GOTERM_MF_DIRECT	transcription factor activity, sequence-specific DNA binding, sequence-specific DNA binding, transcription regulatory region DNA binding,
	MAGE family member A1(MAGEA1)
GOTERM_BP_DIRECT	negative regulation of transcription from RNA polymerase II promoter, transcription, DNA-templated, negative regulation of Notch signaling pathway,
GOTERM_CC_DIRECT	nucleus, cytoplasm, plasma membrane,
GOTERM_MF_DIRECT	protein binding, histone deacetylase binding,
	NOVA alternative splicing regulator 1(NOVA1)
GOTERM_BP_DIRECT	mRNA splicing, via spliceosome, RNA processing, chemical synaptic transmission, locomotory behavior, RNA splicing,regulation of RNA metabolic process,
GOTERM_CC_DIRECT	nucleus, nucleolus, intracellular membrane-bounded organelle,
GOTERM_MF_DIRECT	RNA binding, mRNA binding, poly(A) RNA binding,
	POU class 4 homeobox 1(POU4F1)
GOTERM_BP_DIRECT	negative regulation of transcription from RNA polymerase II promoter, suckling behavior, ventricular compact myocardium morphogenesis, regulation of transcription from RNA polymerase II promoter, transcription from RNA polymerase II promoter, axonogenesis, synapse assembly, mesoderm development, positive regulation of gene expression, cell migration in hindbrain, trigeminal nerve development, central nervous system neuron differentiation, habenula development, neuron projection development, positive regulation of apoptotic process, negative regulation of neuron apoptotic process, positive regulation of transcription from RNA polymerase II promoter, neuron fate specification, sensory system development,peripheral nervous system neuron development, regulation of neurogenesis, proprioception involved in equilibrioception,innervation, positive regulation of cell cycle arrest, regulation of signal transduction by p53 class mediator, negative regulation of transcription elongation from RNA polymerase I promoter,
GOTERM_CC_DIRECT	nuclear chromatin, nucleoplasm, neuron projection,
GOTERM_MF_DIRECT	RNA polymerase II distal enhancer sequence-specific DNA binding, RNA polymerase II transcription factor activity, sequence-specific DNA binding, transcriptional activator activity, RNA polymerase II core promoter proximal region sequence-specific binding, transcriptional activator activity, RNA polymerase II distal enhancer sequence-specific binding,chromatin binding, single-stranded DNA binding, transcription factor activity, RNA polymerase II distal enhancer sequence-specific binding, sequence-specific DNA binding, GTPase binding,
	solute carrier organic anion transporter family member 4C1(SLCO4C1)

GOTERM_BP_DIRECT	multicellular organism development, spermatogenesis, cell differentiation, sodium-independent organic anion transport,
GOTERM_CC_DIRECT	plasma membrane, integral component of plasma membrane, basolateral plasma membrane, extracellular exosome,
GOTERM_MF_DIRECT	sodium-independent organic anion transmembrane transporter activity,

Supplementary Table 7. The primers used in Q-PCR.

SLCO4C1	Forward primer	CAGACATGAAGAGCGCCAAAG
	Reverse primer	AATCAGGCCAGTCAGGGAAC
IZUMO2	Forward primer	CGTGGTCATCGTGGTCTCAT
	Reverse primer	TGCAGCAGGAGTTTTCGGTT
Gapdh	Forward primer	TCACACCAAGTGTCAGGACG
	Reverse primer	CGCCTTCTGCCTTAACCTCA

Supplementary Table 8. Clinical information of cancer samples.

barcode	sex	age	Drug Treatment
MCA 1	male	61	FOLFOX
MCA 2	male	76	capecitabine (Xeloda)
MCA 3	male	62	NULL
MCA 4	famale	67	capecitabine (Xeloda)
MCA 5	famale	72	capecitabine (Xeloda)
MCA 6	famale	71	capecitabine (Xeloda)
MCA 7	male	57	FOLFOX
MCA 8	famale	68	FOLFOX
MCA 9	famale	66	NULL
MCA 10	famale	64	capecitabine (Xeloda)
MCA 11	famale	59	capecitabine (Xeloda)
MCA 12	famale	67	FOLFOX
MCA 13	male	53	FOLFOX
MCA 14	famale	50	capecitabine (Xeloda)
MCA 15	male	59	NULL
MCA 16	male	78	NULL
MCA 17	male	56	NULL
MCA 18	male	55	FOLFOX
MCA 19	famale	79	capecitabine (Xeloda)
MCA 20	famale	79	NULL
MCA 21	male	70	NULL
MCA 22	famale	52	Oxaliplatin
MCA 23	male	51	FOLFOX

Supplementary Table 9. Ct value from q-pcr in 23 clinical samples.

	IZUMO2	MAGEA1	NOVA1	POU4F1	SLCO4C1	Gapdh
MCA 1	21.2807	18.8851	22.2272	20.92933	15.99579	21.73122
MCA 2	24.74783	23.8481	24.07526	23.5759	20.77292	22.97907
MCA 3	20.47353	19.7904	20.36304	21.20656	13.98269	22.05626
MCA 4	21.66257	19.0181	16.91892	22.44433	16.9999	19.23123
MCA 5	22.17938	21.66666	23.48081	22.61432	15.41321	24.69027
MCA 6	23.4627	19.10828	23.46263	21.44453	21.0001	21.2314
MCA 7	20.86937	20.36985	23.26934	22.77899	14.1883	24.23537
MCA 8	20.18949	19.89247	21.73698	21.63366	16.36054	18.74711
MCA 9	16.31971	17.86882	22.99384	21.23036	11.99153	19.45321
MCA 10	20.97392	18.9877	19.52654	19.97768	15.83085	20.42057
MCA 11	18.06733	19.49522	24.47073	22.69279	15.3938	22.42538
MCA 12	18.96714	19.35577	22.27516	22.14325	18.06514	21.05424
MCA 13	19.73353	18.68184	19.04233	19.25841	15.81936	18.69569
MCA 14	17.97098	18.05894	20.50971	19.97168	11.27671	21.31431
MCA 15	22.69149	22.6906	23.95446	24.08643	18.82267	20.59124
MCA 16	15.73483	16.96355	21.87625	20.01548	11.75069	18.21312
MCA 17	19.919	18.19356	18.4443	18.84975	14.586	19.13123
MCA 18	15.96251	17.81798	23.04227	21.00624	13.92239	20.23101
MCA 19	19.25487	19.81127	22.45747	22.4641	19.03538	21.23134
MCA 20	17.13829	18.34815	23.40846	21.53818	14.57399	21.26089
MCA 21	20.52018	20.57807	24.27254	24.29998	19.98587	22.23132
MCA 22	21.24378	18.24666	20.46705	20.39868	17.87598	21.44672
MCA 23	18.54152	23.53904	24.21647	23.83018	18.53573	23.88021
